# A subcompatible rhizobium strain reveals infection duality in *Lotus*

**DOI:** 10.1093/jxb/erz057

**Published:** 2019-02-18

**Authors:** Juan Liang, Andreas Klingl, Yen-Yu Lin, Emily Boul, Jane Thomas-Oates, Macarena Marín

**Affiliations:** 1 Genetics, Faculty of Biology, Ludwig Maximilians University Munich, Germany; 2 Botany, Faculty of Biology, Ludwig Maximilians University Munich, Germany; 3 Department of Chemistry, University of York, UK

**Keywords:** Infection thread, intercellular infection, *Lotus burttii*, Nod factor, nodulation, ‘peg’-like structure, *Rhizobium leguminosarum*, root nodule symbiosis

## Abstract

*Lotus* species develop infection threads to guide rhizobia into nodule cells. However, there is evidence that some species have a genetic repertoire to allow other modes of infection. By conducting confocal and electron microscopy, quantification of marker gene expression, and phenotypic analysis of transgenic roots infected with mutant rhizobia, we elucidated the infection mechanism used by *Rhizobium leguminosarum* Norway to colonize *Lotus burttii*. *Rhizobium leguminosarum* Norway induces a distinct host transcriptional response compared with *Mesorhizobium loti*. It infects *L. burttii* utilizing an epidermal and transcellular infection thread-independent mechanism at high frequency. The entry into plant cells occurs directly from the apoplast and is primarily mediated by ‘peg’-like structures, the formation of which is dependent on the production of Nod factor by the rhizobia. These results demonstrate that *Lotus* species can exhibit duality in their infection mechanisms depending on the rhizobial strain that they encounter. This is especially relevant in the context of interactions in the rhizosphere where legumes do not encounter single strains, but complex rhizobial communities. Additionally, our findings support a perception mechanism at the nodule cell entry interface, reinforcing the idea that there are successive checkpoints during rhizobial infection.

## Teaser

In the rhizosphere, legumes encounter mixed rhizobial communities. *Lotus* species exhibit dual infection mechanisms depending on the rhizobial strain that they encounter.

## Introduction

Legumes engage in a mutualistic interaction with a group of diazotrophic bacteria collectively known as rhizobia. In this interaction, the host plant provides photosynthetic products in exchange for ammonia converted from atmospheric nitrogen by the rhizobia (Oldroyd *et al.*, 2011). This intimate bidirectional nutrient exchange takes place within cells of root organs called nodules. One of the fundamental questions in the field of root nodule symbiosis is how rhizobia enter these plant cells during nodule organogenesis.

The programmes leading to nodule organogenesis and cell infection are distinct, but interconnected (Madsen *et al.*, 2010). Furthermore, the host controls both processes. The infection programme ensures that the rhizobia are guided from the root surface into cells of a dividing nodule primordium in three conceptual steps: (i) crossing of the epidermis; (ii) cortical spreading; and (iii) uptake of rhizobia into plant cells. However, this is attained differently depending on the host legume (Ibáñez *et al*., 2017). For example, in model organisms such as *Medicago truncatula* and *Lotus japonicus*, and crops such as *Pisum sativum*, infection is initiated in epidermal root hairs by the inward growth of plant-made tubular structures called infection threads. Progression of a transcellular infection thread network in developing nodule primordia ultimately leads to the internalization of rhizobia by cells in this tissue (Gage, 2002, 2004; Murray, 2011). The semi-aquatic legume *Sesbania rostrata* is infected under flooding conditions through physical cracks in the root epidermis, for example at lateral root emergence sites (Ndoye *et al.*, 1994). Here, proliferating bacteria accumulate in intercellular infection pockets that give rise to trans- and intercellular infection threads (Ndoye *et al.*, 1994). Some subtropical legumes, such as *Neptunia natans* and *Aeschynomene afraspera*, also become infected through cracks, but the uptake into plant cells occurs directly from intercellular accumulations of bacteria, in the absence of infection threads (James *et al.*, 1992; Subbarao *et al.*, 1995; Bonaldi *et al.*, 2011). As a final example, there are plants such as *Lupinus albus*, in which bacteria cross the epidermis intercellularly, but are also directly internalized from intercellular accumulations (Gonzalez-Sama *et al.*, 2004). Thus, conceptually, there are infection thread-dependent and -independent infection mechanisms controlled by epidermal and nodule primordium programmes (Madsen *et al.*, 2010).

Genetic studies using gain-of-nodulation mutants have elegantly illustrated these different infection programmes in *L. japonicus* (Madsen *et al.*, 2010). *Mesorhizobium loti* infects *L. japonicus* wild-type plants via infection threads (van Spronsen *et al.*, 2001). However, it invades *nfr1-1 nfr5-2 snf1* triple mutant plants in a process resembling the epidermal thread-independent crack-entry infection observed in *S. rostrata* (Ndoye *et al.*, 1994; Madsen *et al.*, 2010). Another major discovery of this work was that a *M. loti nodC* mutant strain infects *nfr1-1 nfr5-2 snf1* triple mutants at low frequency in the absence of epidermal and transcellular infection threads (Madsen *et al.*, 2010). These results demonstrate that *Lotus* possesses a genetic repertoire allowing multiple types of infection. However, whether this also occurs in wild-type plants and natural *Lotus* strains has not yet been conclusively demonstrated.

The existence of an infection mechanism mediating the direct entry into plant cells from the intercellular space (independent of epidermal and transcellular infection threads) at high frequency would constitute an invaluable tool to study this key step in the evolution of root nodule symbiosis. In this work, we investigated whether wild-type *Lotus* can be infected by an infection thread-independent mechanism, using molecular approaches and detailed microscopy. We discovered that a natural *Lotus* isolate infects different wild-type *Lotus* plants utilizing an epidermal and transcellular infection thread-independent mechanism at high frequency. Moreover, the penetration into plant cells is primarily mediated by ‘peg’-like structures, the formation of which is dependent on the production of Nod factors by the rhizobia.

## Materials and methods

### Bacterial strains and growth conditions

The bacterial strains used in this study are listed in Supplementary Table S1 at *JXB* online. Rhizobia cultures were grown for 2 d at 28 °C in different media depending on the experiment. For nodulation and infection assays, rhizobia were grown in tryptone yeast extract (TY) broth (Beringer, 1974). For gene expression analyses, strains were grown in yeast mannitol broth (YMB) (Vincent, 1970). Finally, for Nod factor production, *Rhizobium leguminosarum* (*Rl*) Norway was grown in TY broth and then subcultured in modified B^−^ medium (modified from Spaink *et al.*, 1992). As the carbon source, 5 g l^−1^ mannitol and 5 g l^−1^ sodium gluconate were used. For *nod* gene induction, the medium was supplemented with 1 µM naringenin for 2 d. *Agrobacterium* strains used in the hairy root transformation experiment were grown for 1 d at 28 °C in yeast extract broth (YEB) (Vervliet *et al.*, 1975). The *Escherichia coli* strains used in the conjugation assay were grown for 1 d at 37 °C in Luria Bertani (LB) broth. The following antibiotic concentrations were used: tetracycline (Tc, 2–10 µg ml^−1^); gentamicin (Gm, 25 µg ml^−1^); kanamycin (Km, 50 µg ml^−1^); streptomycin (Sm, 500 µg ml^−1^); rifampicin (Rf, 50 µg ml^−1^); and carbenicillin (Cb, 50 µg ml^−1^).

### Plant growth and inoculation conditions

*Lotus burttii* B-303 (seed bag numbers: 91091, 91101, and 91103) and *Lotus japonicus* MG-20 (seed bag number: 92147) seeds were surface sterilized with a 1.2% NaClO solution, rinsed, and soaked in water at room temperature for 2 h. Seeds were then transferred to 1/2 B5 medium agar plates and kept at 24 °C for 3 d in the dark and 3 d under a long-day photoperiod (16 h:8 h, light:dark). For shoot growth, nodulation, and infection quantification, three independent time-course experiments were conducted with 20 plants per condition and per time point. Six-day-old seedlings were transferred to sterile jars containing 300 ml of a sand:vermiculite mixture supplemented with 40 ml of FAB medium. After 2 d, each plant was inoculated with 1 ml of bacterial suspension (*A*_600_=0.005). For root hair phenotypic analysis and infection thread quantification, four independent experiments were conducted with 20 plants per condition. Six-day-old seedlings were gently placed over sterile filter paper (Whatman) on square Petri plates containing FAB medium. After 2 d, vertically grown plants were inoculated with bacterial suspensions (*A*_600_=0.05), covered with a second sheet of sterile filter paper, and incubated under a long-day photoperiod. Plants were inspected 1, 2, and 3 weeks post-inoculation (wpi).

### Hairy root transformation

To overexpress SYMRK in the roots of *L. burttii* plants, the roots of 6-day-old seedlings were cut and the remaining hypocotyl regions were dipped into *Agrobacterium rhizogenes* AR1193 (Stougaard *et al.*, 1987) suspensions carrying the relevant plasmids (Supplementary Table S1). Transformed plants were grown on B5 medium in the dark at room temperature for 3 d and then moved to a long-day photoperiod at 24 °C. After 2 d, plants were transferred to B5 medium supplemented with cefotaxime (300 µg ml^−1^) to clear the *Agrobacterium*. After 23 d, seedlings were screened for transformation, using a green fluorescent protein (GFP)-based transformation marker. Transformed plants were transferred to closed sterile jars containing 300 ml of a sand:vermiculite mixture supplemented with 40 ml of FAB medium. After 2 d, each plant was inoculated with a 1 ml bacterial suspension (*A*_600_=0.01) and grown under a long-day photoperiod. Plants were harvested 9 wpi and phenotypically analysed. Three independent experiments were conducted with at least 20 plants per condition.

### Histological staining and microscopy

To inspect nodule colonization, samples were fixed with a 2.5% glutaraldehyde solution in 0.5 M potassium phosphate buffer and progressively dehydrated in 30, 50, 70, and 100% ethanol solutions for 1 h each. Nodules were then embedded in a Technovit 7100 resin (Heraeus Kulzer) according to the manufacturer’s instructions, and 2 μm thin sections were cut with an RM2125 RT rotary microtome (Leica Biosystems). Sections were placed on glass slides and dried at 60 °C for 30 min. Dried sections were stained with a 1% toluidine blue and 0.2% methylene blue mixed solution for 30–60 s and rinsed with water until the background cleared. Stained sections were inspected on a DM6 B upright microscope (Leica Microsystems) equipped with ×5, ×10, and ×40 dry objectives and a ×20 oil/water immersion lens.

For fluorescence microscopy analyses of nodule colonization, samples were fixed with a 4% formaldehyde solution in 50 mM PIPES buffer by 30 min vacuum infiltration and then kept at room temperature for 45 min. The fixed samples were embedded in 6% low melting agarose (Carl Roth), and semi-thin sections (40–50 µm) were cut with a VT1000S vibratome (Leica Biosystems) at speed five and frequency five. Nodule sections were counterstained with a fresh 0.01% calcofluor white solution for 10 min. To visualize the colonization of *Rl* Norway*∆nodC* in spontaneously induced nodules, sections were additionally stained with a 20 µM propidium iodide (PI) solution for 10 min. For the rhizobia viability assay, fresh nodules were sectioned and stained with a Live/Dead BacLight Bacterial Viability kit (3.34 µM SYTO9 and 20 µM PI; Invitrogen) for 10 min at room temperature. Agarose semi-thin sections were observed using a TCS SP5 confocal microscope (Leica Microsystems) equipped with a ×20 HCX PL APO water immersion lens. Calcofluor white was excited with UV and the emission was detected at 405–450 nm. GFP, SYTO9, and PI were excited with an argon laser line at 488 nm and the emissions were detected at 500–550, 500–550, and 600–650 nm, respectively. *Ds*Red was excited with a diode pumped solid-state laser at 561 nm and detected at 600–650 nm.

### Quantitative analysis of images

To quantify the percentage of nodule colonization, an area comprising the total inner tissue of the nodule was manually defined using Fiji v.2.0.0-rc-59/1.51j (Schindelin *et al.*, 2012). The colonized area was calculated for each section by defining a signal threshold and masking the regions below it. The average percentage of 1–3 sections per nodule and at least 5–6 nodules per condition were used for the calculations.

### Electron microscopy

Root nodules were pre-fixed in 50 mM PIPES buffer (fixation buffer 1) containing 2.5% glutaraldehyde. The nodules were cut into smaller pieces in this fixation buffer and afterwards transferred to 50 mM cacodylate buffer containing 2 mM MgCl_2_ (fixation buffer 2) and 2.5% glutaraldehyde for complete fixation overnight at 4 °C. After washing the samples four times (10, 30, 30, and 50 min) with fixation buffer 2 without glutaraldehyde, post-fixation with 1% osmium tetroxide was carried out for 1.5 h. Afterwards, they were washed again twice with fixation buffer 2 (without glutaraldehyde) and four times with double-distilled water (45, 35, 30, and 30 min). The dehydration of the samples was achieved in a graded acetone series before infiltration and embedding in Spurr’s resin. The thin sections of embedded samples were post-stained with lead citrate for 2 min and investigated on a Zeiss EM 912 transmission electron microscope with an integrated OMEGA filter. The acceleration voltage was set to 80 kV and the images were recorded with a Tröndle 2k×2k slow-scan CCD camera.

### Quantitative RT–PCR

For the quantification of gene expression, materials were collected from whole root systems, nodules, and rhizobia pellets, and then snap-frozen in liquid nitrogen. All samples were lysed with an MM40 tissue lyser (Retsch). Total RNA was extracted with the Spectrum™ Plant Total RNA kit (Sigma-Aldrich) according to the manufacturer’s instructions. To eliminate DNA contamination, DNase I (Ambion) treatment was conducted, and then plant and bacterial samples were analysed by PCR using *ATP-synthase* (*ATP*) and *ubiquitin* primers, and *initiation factor 1* (*IF-1*) primers, respectively (Supplementary Table S2). RNA integrity was verified on an agarose gel. Superscript III reverse transcriptase (Thermo Fisher) was used to synthesize first-strand cDNA using 270 ng of total RNA. Quantitative reverse transcription–PCR (qRT–PCR) was performed on a 384-well plate with the Quantstudio5 system (Thermo Fisher) and using the Evagreen Master mix (Metabion) according to the manufacturer’s instructions. The reaction was performed with a 1:10 (v/v) dilution of the cDNA, with 0.3 µM of each primer in a total reaction volume of 7 µl.  The thermal cycler conditions were: 95 °C 2 min, 40 cycles of 95 °C 30 s, 58 °C 30 s, and 72 °C 20 s, followed by dissociation curve analysis. At least five biological replicates and 2–3 technical replicates were included for the quantification of each gene. Normalization of plant and rhizobia genes was performed using the *ATP* and *IF-1* housekeeping genes, respectively. All qRT–PCR primers used in this work are listed in Supplementary Table S3.

### Nod factor isolation

The Nod factors were extracted from the supernatant of a 3 litre *Rl* Norway culture with *1*-butanol (300 ml l^−1^ culture). The Nod factors were collected by evaporating the butanol phase in a Hei-VAP Value Rotary Evaporator (Heidolph Instruments). The dried extract was redissolved in 3.5 ml of 60% aqueous acetonitrile (ACN) (v:v) by shaking for 18 h. A 1.5 ml aliquot of the resulting solution was diluted by addition of ACN to a final concentration of 20% (v/v) aqueous ACN and loaded onto a primed C18 solid phase extraction cartridge (Supelclean ENVI-18, 1 g bed weight; Sigma-Aldrich). The cartridge was washed with 5 ml of 20% (v:v) aqueous ACN and the Nod factors were eluted with 5 ml of 45% ACN, followed by 5 ml of 60% ACN. The two eluted fractions were collected separately and dried under vacuum, prior to reconstitution in 0.7 ml of 60% ACN for HPLC fractionation.

### HPLC fractionation of Nod factors

The 45% and 60% SPE fractions were each diluted to a final concentration of 20% ACN. A 1.5 ml aliquot of the resulting solution was injected onto an Agilent Technologies 1200 series HPLC instrument fitted with a reversed phase column (Waters SymmetryShield RP18, 5 µm particles, 4.6×250 mm, with guard column) eluted at 1 ml min^–1^, using UV detection at 205 nm. The column was eluted using the following gradient: 20 min isocratic at 20% ACN, linear elution from 20% to 60% ACN over 20 min, linear gradient 60% to 90% over 0.5 min, isocratic at 90% ACN for 4.5 min, and then re-equilibrated at 20% ACN for 5 min. Fractions of 1 min were collected and dried under vacuum.

### Nod factor structure determination

Mass determination of the Nod factors in the HPLC fractions was carried out using a Bruker 9.4 T solariX HR Fourier-transform ion cyclotron resonance instrument in the York Centre of Excellence in Mass Spectrometry (CoEMS). The instrument was operated in the positive ion mode using a matrix-assisted laser desorption/ionization (MALDI) source. HPLC fractions were redissolved in 50 µl of 80% ACN, and 2 µl of this sample solution was mixed with 2 µl of MALDI matrix solution (2,5-dihydroxybenzoic acid; 7 mg in 500 µl of 80% ACN); 0.8 µl of this mixture was spotted onto a ground steel MALDI target plate and allowed to air dry. Spectra were acquired by irradiating the dried sample spots with the laser (Smartbeam: Nd:YAG 355 nm) set at 35% laser power and a frequency of 500 Hz. Fragmentation was generated using collision-induced dissociation (CID) with collision voltage settings varied between 25 V and 35 V, and product ion spectra were recorded. Alternatively, CID product ion spectra were recorded using static nanoelectrospray ionization in the positive ion mode with a Thermo Scientific Orbitrap Fusion in CoEMS. Samples were dissolved in 50 µl of 50% ACN, and 2 µl was transferred to the electrospray tip (made in-house). Higher energy collisional dissociation spectra were recorded using collision ‘energy’ settings between 20V and 30 V. Nod factor structures were determined from interpretation of the product ion spectra obtained on the two instruments.

### Conjugation

The GFP-expressing plasmid pFAJ-GFP and the suicide replacement plasmid pK19MOBSACB (Supplementary Table S1) were introduced into rhizobia by conjugation using *E. coli* ST18 (Thoma and Schobert, 2009) as donor strain. The donor and acceptor strains (*A*_600_=1) were mixed in a 10:1 ratio. The mixtures were placed on TY plates and incubated at 28 °C. After 24 h, bacteria were suspended and grown on selective TY plates.

### Generation of the *Rl* Norway*∆nodC* deletion mutant

The two-step homologous recombination method described previously (Sant’anna *et al.*, 2011) was used to generate deletion mutants in *Rl* Norway. Two 500 bp fragments flanking the *nodC* gene were amplified by PCR and cloned into the suicide vector pK19MOBSACB (Supplementary Table S1). The plasmid was delivered into *Rl* Norway by conjugation. The first recombination event was selected on TY medium supplemented with Km. Positive colonies were verified by PCR using plasmid- and genome-specific primers (Supplementary Table S2). The second recombination event was counter-selected on TY medium containing 10% sucrose. Mutants were verified by PCR and sequencing using primers annealing upstream and downstream of the flanking fragments (Supplementary Table S2).

### Statistical analyses

All statistical analyses were performed in R-studio by using ANOVA and Tukey honest significant difference (TukeyHSD) methods.

## Results

### *Rhizobium leguminosarum* Norway induces ineffective nodules in wild-type *Lotus burttii*

**Fig. 1.**
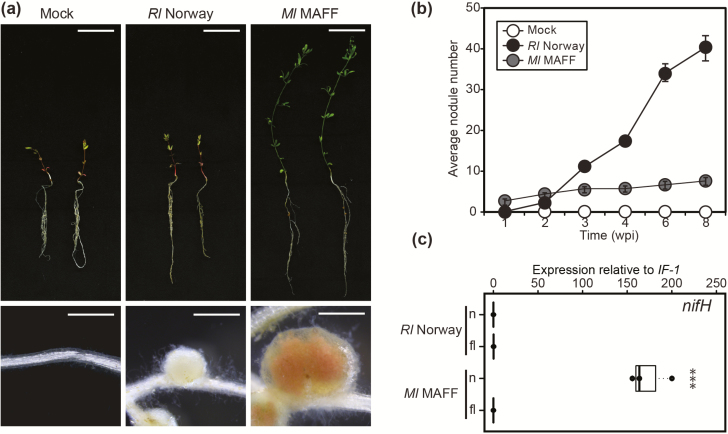
*Rhizobium leguminosarum* Norway induces ineffective nodules in *Lotus burttii*. (a) Images of shoot (upper panel) and nodule (lower panel) phenotypes exhibited by representative *L. burttii* plants 6 weeks after mock treatment, or inoculation with *Rl* Norway and *Mesorhizobium loti* MAFF303099. Scale bars: (upper panel) 1 cm; (lower panel) 1 mm. (b) Time-course quantification of the average nodule number per plant. Three independent experiments were conducted with 20 plants per condition and per time point. Error bars indicate the SDs. (c) Quantification of *nifH* transcript abundance by qRT–PCR. Total RNA was extracted from *L. burttii* nodules (n) induced by *Rl* Norway and *Ml* MAFF at 4 wpi, and from free-living (fl) rhizobia grown in liquid culture. Relative transcript expression was normalized against the housekeeping gene *Initiation factor-1*. Each dot represents one independent biological replicate. The bold black line and the box represent the median and the interquartile range, respectively. The statistical analysis was performed by ANOVA; ***P<0.001.

*Lotus burttii* is a *Lotus* species originally identified in West Pakistan (Borsos *et al.*, 1972) and is nodulated by a wide range of rhizobia including *Mesorhizobium loti* MAFF303099 (*Ml* MAFF) (Gossmann *et al.*, 2012), *Sinorhizobium fredii* HH103 (Acosta-Jurado *et al.*, 2016), and *Rhizobium leguminosarum* (*Rl*) Norway, a natural *Lotus* isolate (Gossmann *et al.*, 2012). Interestingly, *Rl* Norway infects *L. burttii* nodules apparently in the absence of epidermal infection threads (Gossmann *et al.*, 2012). We characterized the symbiotic interaction between this strain and *L. burttii*, and compared it with the interaction with *Ml* MAFF. We inoculated *L. burttii* plants under axenic conditions and analysed growth and nodule organogenesis in time-course experiments. *Ml* MAFF promoted shoot growth and induced pink nodules on the roots of *L. burttii* (Fig. 1a). In comparison, *Rl* Norway induced a larger number of nodules (Fig. 1b; Supplementary Fig. S1a). However, these nodules were ineffective, as the inoculated seedlings had stunted shoots and their leaves were pale yellow, a sign of nitrogen starvation (Fig. 1a; Supplementary Fig. S1b).

To validate further the lack of nitrogen fixation in *Rl* Norway-induced nodules, we determined by qRT–PCR the relative expression of the rhizobial *nifH* gene. This gene encodes a nitrogenase subunit that is essential for nitrogen fixation and is markedly induced in nitrogen-fixing nodules (Uchiumi *et al.*, 2004). The *nifH* gene of *Ml* MAFF was induced in nodules in comparison with free-living conditions. In contrast, *Rl* Norway exhibited no induction of *nifH* under the same conditions (Fig. 1c). This shows that *Rl* Norway induces ineffective nodules in *L. burttii*.

### *Rl* Norway induces a distinct early symbiotic response

**Fig. 2.**
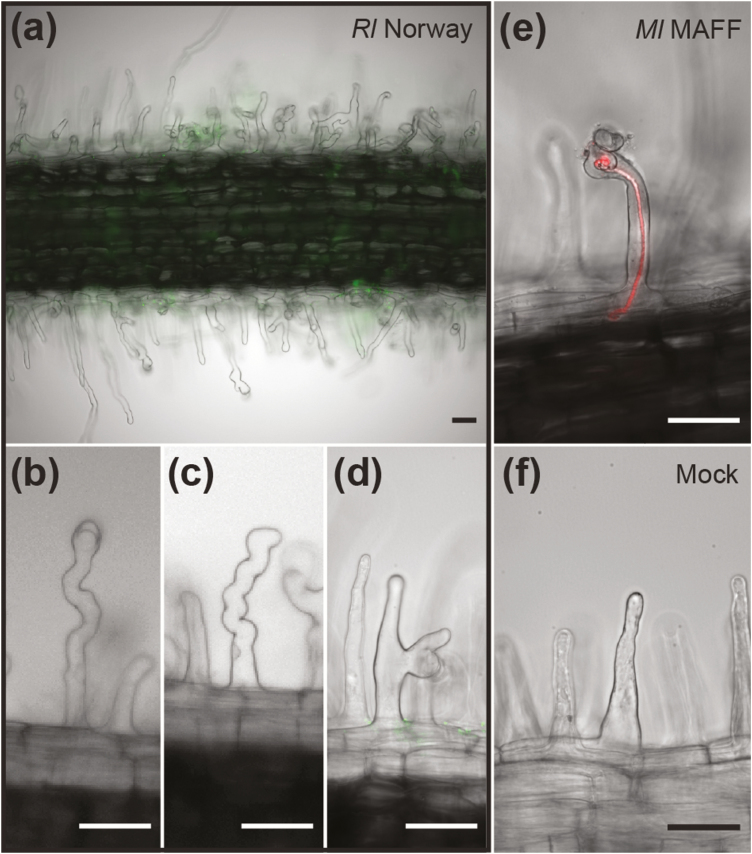
*Rhizobium leguminosarum* Norway induces root hair deformations in *Lotus burttii*. (a) Overview of a root segment colonized by *Rl* Norway–GFP that displays no epidermal infection threads, but massive root hair deformation, including different degrees of swelling (b, c), and branching (d). Representative micrographs of an infection thread induced by *Mesorhizobium loti* MAFF303099-*Ds*Red (e) and of root hairs upon mock treatment (f). Four independent experiments were conducted with 20 plants per condition on the square Petri plates. Scale bars=50 µm.

To investigate the mechanism by which *Rl* Norway infects *Lotus*, we visually inspected the root hairs of *L. burttii* plants grown on plates. The roots inoculated with *Rl* Norway showed extensive root hair swelling, and branching, but only rarely curling (Fig. 2a–d). In contrast to the responses to *Ml* MAFF, the root hair deformations were not confined to the susceptible zone, but extended throughout the majority of the root. Such an unrestricted response has been observed in roots of *L. japonicus* and *Glycine max* after Nod factor application (Niwa *et al.*, 2001; Duzan *et al.*, 2004) or in the *L. japonicus symrk-3* mutant upon inoculation with *M. loti* R7A (Stracke *et al.*, 2002).

In accordance with previous reports (Gossmann *et al.*, 2012), no epidermal infection threads were observed upon inoculation with *Rl* Norway under the experimental conditions tested. We analysed >100 plants grown on plates for a period of 1–3 weeks. Infection threads were also absent upon inoculation of *L. japonicus* MG-20. In contrast, *L. burttii* and *L. japonicus* MG-20 plants exhibited only minor root hair deformations 1 week after inoculation with *Ml* MAFF, but developed an average of 7±3 and 17±6 infection threads per plant, respectively (Fig. 2e).

**Fig. 3.**
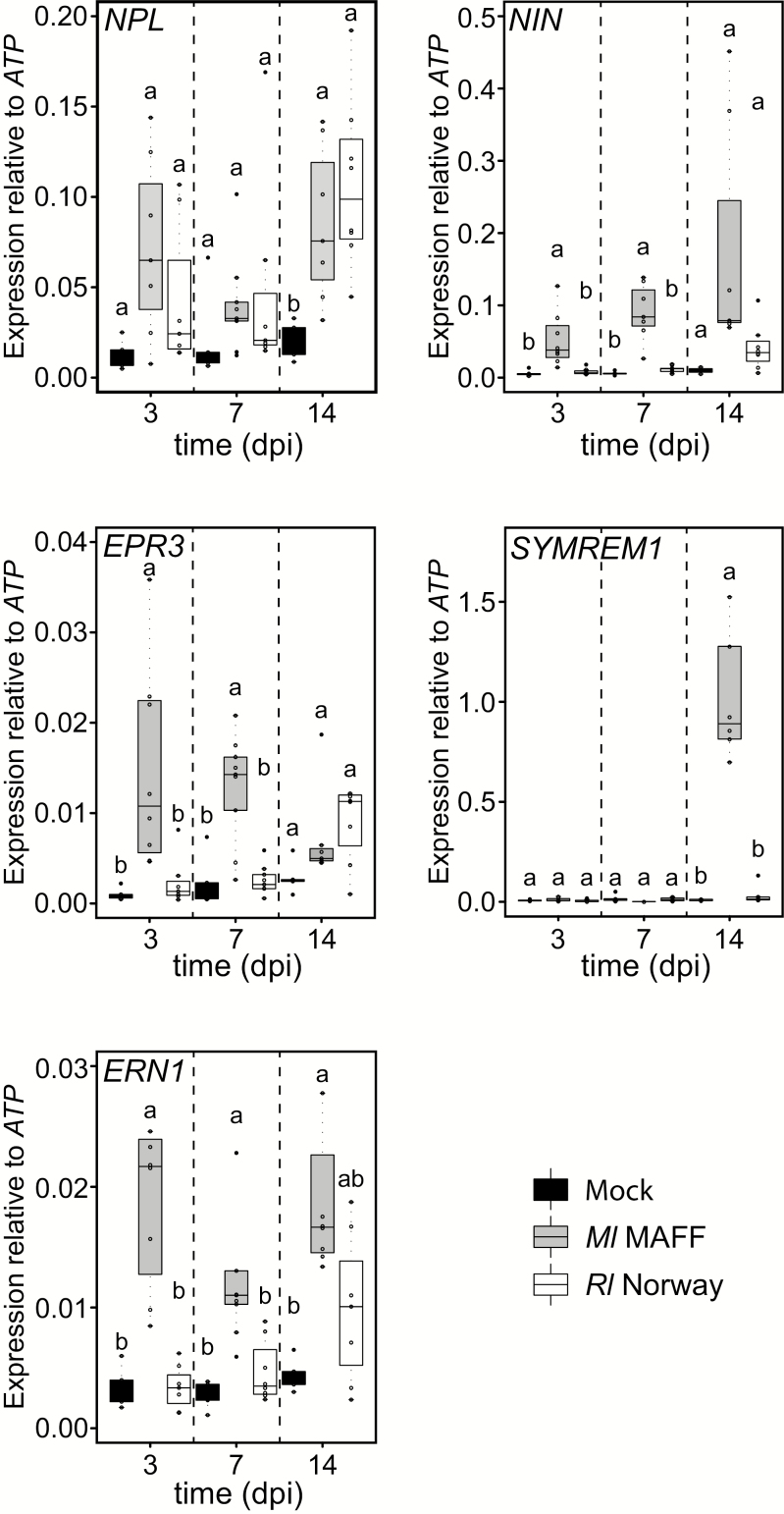
Gene expression analysis of *Lotus burttii* roots upon rhizobial inoculation. Quantification of *NPL*, *NIN*, *ERN1*, *EPR3*, and *SYMREM1* transcript abundance by qRT–PCR. Total RNA was extracted from *L. burttii* whole root systems after mock treatment and after 3 d, 1 week, and 2 weeks post-inoculation with *Rhizobium leguminosarum* Norway and *Mesorhizobium loti* MAFF303099. Relative transcript expression levels were normalized against the housekeeping gene *ATP-synthase*. Each dot represents one independent biological replicate. The bold black line and the box represent the median and the interquartile range, respectively. The statistical analysis was performed for each time point using ANOVA and TukeyHSD methods. Lower case letters indicate significance groups within each time point.

To determine molecular responses induced by *Rl* Norway, we quantified by qRT–PCR the expression of symbiotic marker genes involved in infection, such as *Nodule INception* (*NIN*), *Nodulation Pectate Lyase* (*NPL*), *ExoPolysaccharide receptor 3* (*EPR3*), *ERF Required for Nodulation 1* (*ERN1*), and *SYMbiotic REMorin 1* (*SYMREM1*) at 3, 7, and 14 days post-inoculation (dpi). *Rl* Norway induced distinct gene expression compared with *Ml* MAFF (Fig. 3). At 3 dpi, a time point at which nodules had not developed in any of the conditions, only roots inoculated with *Ml* MAFF significantly induced the expression of *NIN*, *ERN1*, and *EPR3*. *NIN* induction was slightly delayed in *Rl* Norway-inoculated roots. This coincided with a delayed nodulation phenotype exhibited in these roots (Fig. 1b). A similar pattern was observed for *ERN1* and *EPR3* (Fig. 3). Most strikingly, at 14 dpi, *SYMREM1* expression was almost 30-fold higher in *Ml* MAFF-inoculated roots compared with *Rl* Norway-inoculated roots (Fig. 3). These quantitative differences in the expression of infection marker genes at 3 dpi could explain the absence of epidermal infection threads. These results indicate that *Rl* Norway induces a distinct response in *L. burttii* compared with *Ml* MAFF.

### *Rl* Norway induces intercellular ‘peg-like’ structures

**Fig. 4.**
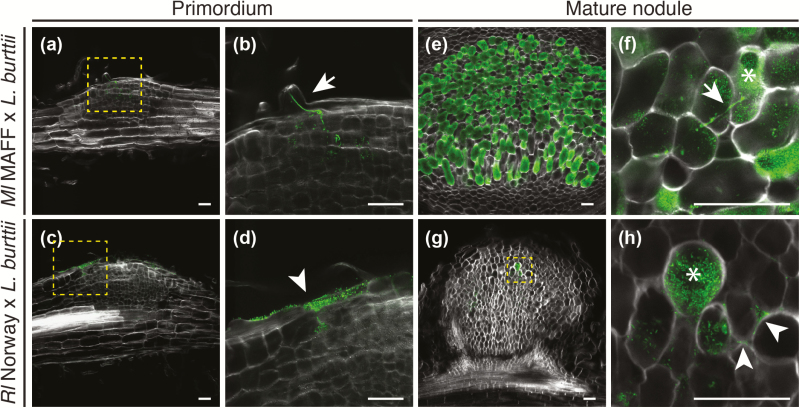
*Rhizobium leguminosarum* Norway colonizes *Lotus burttii* nodules in the absence of transcellular infection threads. Representative confocal laser scanning micrographs of nodule semi-thin sections (50 µm) counterstained with calcofluor white (white) show that (a, b) *Mesorhizobium loti* MAFF303099–GFP bacteria invade the nodule primordium at 5 dpi through epidermal infection threads (b, arrow), while *Rl* Norway–GFP bacteria (c, d) invade the nodule primordium at 11 dpi in the absence of epidermal infection threads (d, arrowhead). (e, f) *Ml* MAFF–GFP bacteria fully colonize the nodule (e) and induce transcellular infection threads (f, arrow) at 3 wpi. In contrast, (g, h) *Rl* Norway–GFP bacteria partially colonize the nodule inter- (h, arrowhead) and intra- (h, asterisk) cellularly at 4 wpi in the absence of transcellular infection threads. The images shown here are representative of 20 primordia and 20 nodules infected by *Rl* Norway, and 5 primordia and 7 nodules infected by *Ml* MAFF. Scale bars=50 µm.

The differential expression of infection marker genes and the absence of infection threads suggested that *Rl* Norway utilizes an infection mechanism distinct from that of *Ml* MAFF to colonize *Lotus*. To investigate this, we sectioned nodules in different developmental stages and visualized their colonization by confocal microscopy using fluorescently tagged strains and TEM. Upon *Ml* MAFF inoculation, infection threads were visible on top of the growing primordia, and underlying cells were infected (Fig. 4a, b). In contrast, *Rl* Norway accumulated on top of empty nodule primordia at sites in which the epidermis had been disrupted due to the nodule emergence (Fig. 4c, d). Structures reminiscent of infection pockets formed at these sites (Supplementary Fig. S2a). This suggests that *Rl* Norway crosses the epidermis through cracks induced by the emergence of nodule primordia and not necessarily at lateral root emergence sites. Accordingly, nodules formed along the complete root system and not preferentially at lateral root bases (Supplementary Fig. S1a).

**Fig. 5.**
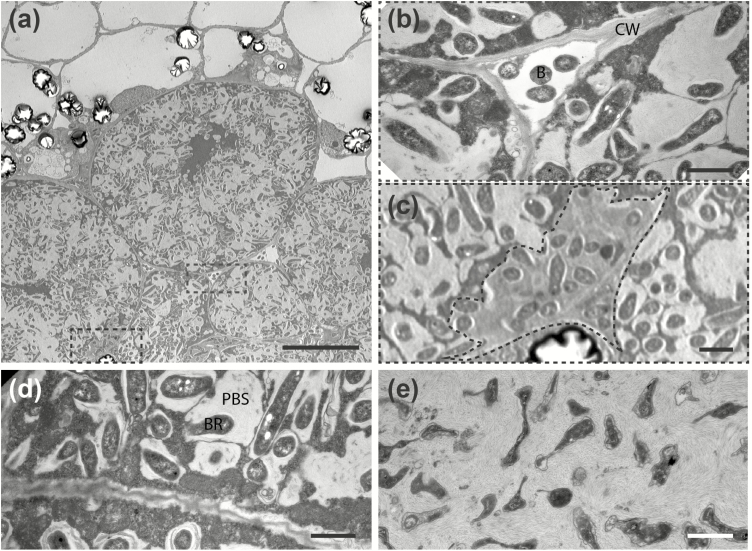
*Rhizobium leguminosarum* Norway enters *Lotus burttii* root nodule cells through ‘peg’-like structures and forms symbiosomes. TEM micrographs of nodule sections infected by *Rl* Norway at 4 wpi. (a) Overview displays intact plant cells infected with rhizobia. Magnifications show: (b) bacteria (B) colonizing the intercellular space, and (c) bacteria surrounded by a dense matrix entering a cell from the intercellular space (‘peg’-like structure surrounded by a dashed line). (d) A nodule cell contains symbiosomes with enlarged peribacteroid spaces (PBS) and elongated bacteroids (BR). (e) Bacteria undergoing degradation. CW, cell wall. Scale bars: (a), 10 µm; (b–e), 1 µm.

At 3 wpi, *Ml* MAFF induced fully developed nodules that were largely colonized (nodule colonization = 67.1 ± 13.5%) and contained transcellular infection threads (Fig. 4e, f). In contrast, *Rl* Norway infected cells intracellularly (nodule colonization = 1.4 ± 0.7%), but induced no transcellular infection threads in >35 sectioned nodules (Fig. 4g, h; Supplementary Fig. S2b, c). We observed in 100% of the nodules analysed intercellular *Rl* Norway accumulations (Fig. 4h; Supplementary Fig. S2d). For a more detailed view, we conducted TEM, which also showed intercellular accumulations (Fig. 5a, b). In 40% of the agarose sections, cells contained structures with densely packed bacteria (Supplementary Fig. S2e). These structures were reminiscent of ‘peg-like’ structures, which have been described previously in *Aeschynomere afraspera* (Bonaldi *et al.*, 2011) and *Lupinus albus* (Gonzalez-Sama *et al.*, 2004). To describe these structures unequivocally, we conducted TEM of ultra-thin nodule sections. A dense material surrounded invading intercellular bacteria (Fig. 5c). These results suggest that cell invasion is mediated via ‘peg’-entry.

*Rl* Norway invaded intact plant cells and formed symbiosomes surrounded by a peri-bacteroid membrane (Fig. 5a). However, infected cells exhibited signs of early senescence, such as disorganized nuclei (Fig. 5a). Furthermore, the symbiosomes had an enlarged peri-bacteroid space, and a polymeric material surrounded the bacteroids (Fig. 5d). At 4 wpi, symbiosome integrity was disrupted and bacteroids were partially degraded (Fig. 5e). To investigate the viability of bacteria, we conducted live/dead staining using PI and SYTO9, which label dead and living bacteria, respectively. *Ml* MAFF bacteria were viable at least up to 6 wpi (Supplementary Fig. S3a, b), in contrast to a fraction of *Rl* Norway bacteria that died as early as 4 wpi (Supplementary Fig. S3c, d).

### The ‘peg’-like infection of SYMRK-induced spontaneous nodules is Nod factor dependent

The Nod factors produced by *M. loti* induce root hair deformations and cortical cell divisions in *Lotus* (Niwa *et al.*, 2001) and are essential for epidermal infection thread formation (Madsen *et al.*, 2010). However, their role in cell entry has not been thoroughly studied. To investigate the role of the Nod factors in the formation of the ‘peg-like’ structures induced by *Rl* Norway, we generated in this strain an in-frame deletion of the *nodC* gene, which encodes the *N*-acetylglucosaminyl transferase responsible for the synthesis of the Nod factor backbone. The *Rl* Norway *nodC* gene is located in a cluster resembling the nod operon of *R. leguminosarum* biovar *viciae* 3841 (Liang *et al.*, 2018) (Supplementary Fig. S4*a*). Consequently, the Nod factors produced by *Rl* Norway resemble the factors produced by other *R. leguminosarum* strains (D’Haeze and Holsters, 2002) (Supplementary Table S4).

**Fig. 6.**
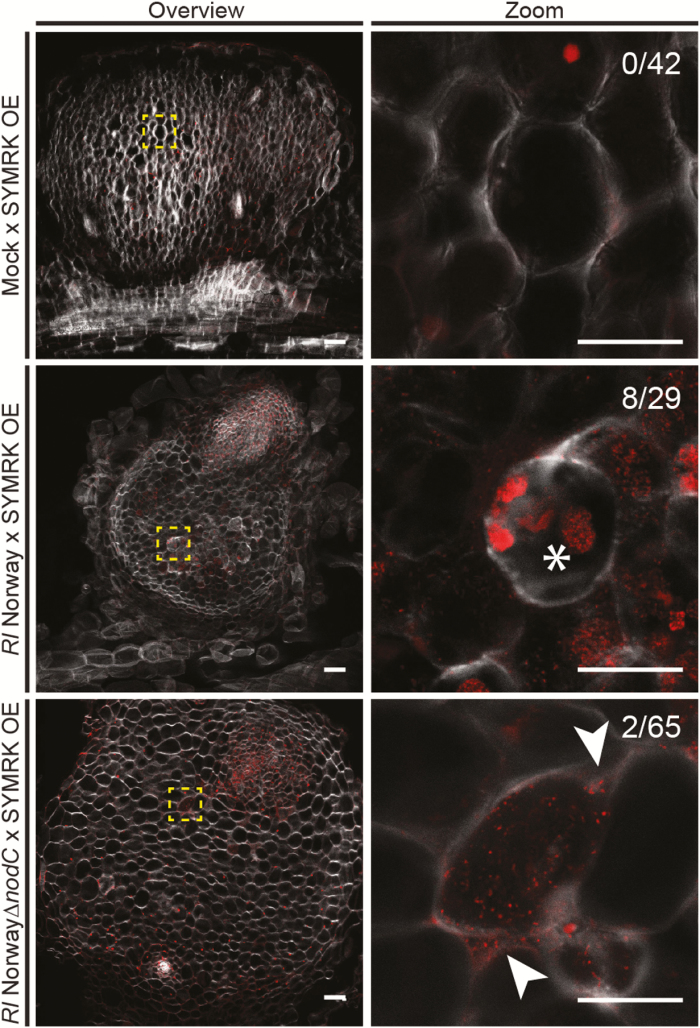
Infection of spontaneously induced nodules in the absence of Nod factors. Hairy roots of *Lotus burttii* transformed with *pUBi:SYMRK-mOrange* were analysed 9 weeks after mock treatment, and inoculation with *Rhizobium leguminosarum* Norway and *Rl* Norway*ΔnodC.* Semi-thin sections (50 µm) of nodules were incubated with calcofluor white and propidium iodine that stain the plant cell wall and bacteria (and plant nuclei), respectively. Confocal laser scanning micrographs show that spontaneously generated nodules are induced even in the absence of rhizobia. *Rl* Norway colonizes nodule cells, and dense bacterial accumulations reminiscent of ‘peg’-like structures (asterisk) are formed. In contrast, nodule cells infected by *Rl* Norway*ΔnodC* do not exhibit these structures. Arrowheads indicate intercellular accumulations. Three independent experiments were conducted with at least 20 plants per condition. Representative micrographs are shown for each condition. Fractions indicate the number of nodules with detectable rhizobial infection per total nodule number for one of the experiments. Scale bars=50 µm.

The deletion in *nodC* abolished the capacity of *Rl* Norway to induce root hair deformations and nodule organogenesis in *L. burttii* (Supplementary Fig. S4b–d). To study the infection of nodule cells, we induced spontaneous nodules by overexpressing the SYMbiotic Receptor-like Kinase SYMRK in transgenic roots (as described in Ried *et al.*, 2014), and inoculated them with *Rl* Norway wild type or *nodC* mutant. Spontaneous nodules only developed in SYMRK transgenic roots, and not in roots transformed with the empty vector. These nodules were excised, fixed, and sectioned. Nodule sections were stained with calcofluor white and PI to visualize the cell wall and bacteria, respectively. Wild-type *Rl* Norway colonized 28.3% of the sectioned nodules. This contrasts with the 100% colonization rate of *Rl* Norway-induced nodules. Approximately 20–50% of the infected cells exhibited ‘peg’-like structures (Fig. 6). In contrast, the *nodC* mutant colonized only 2% of the nodules analysed. The colonization of these nodules was mostly restricted to regions with active cell division, as indicated by smaller plant cell size and multiple nuclei (Fig. 6). In all sections analysed, although infected cells were present, no ‘peg’-like structure was observed with the *nodC* mutant strain. This result suggests that these structures are induced upon Nod factor production and supports a perception mechanism at the interface of nodule cell entry.

## Discussion

Bacterial entry into nodule cells is one of the key steps in the evolution of the root nodule symbiosis. Independent of the infection mechanism, a common feature is the formation of structures that mediate internalization. These are either transcellular (infection threads) (Gage, 2002, 2004) or intercellular (‘peg’-like structures) (Gonzalez-Sama *et al.*, 2004; Bonaldi *et al.*, 2011). The presence of a matrix material in these structures has been proposed as one of the unifying features allowing the intracellular uptake of bacteria into plant cells (Parniske, 2018). Here we describe that *Lotus* allows cell colonization through either transcellular infection threads or ‘peg’-like structures depending on the rhizobial strain encountered.

### Duality in symbiotic infection

Most legumes studied so far are predominantly infected by one infection mechanism. However, duality in infection has been documented. *Sesbania rostrata*, a robinioid plant like *Lotus*, exhibits dual infection behaviour depending on the growth conditions (Goormachtig *et al.*, 2004). Upon flooding, *Sesbania* represses the growth of root hairs, and thus infection threads are not formed. Rhizobia then exploit lateral root bases as entry points (Ndoye *et al.*, 1994). Similar behaviour was described for *Lotus pedunculatus.* Bacteria infect enlarged epidermal cells and accumulate intercellularly in nodules formed on adventitious roots of flooded plants (James and Sprent, 1999). Another example is the ineffective strain NZP2213 that induces pseudo-nodules on *L. pedunculatus* roots. These organs are colonized intercellularly, but no cell infection is observed (Pankhurst *et al.*, 1979). Genetic manipulation of *L. japonicus* leads to differential colonization modes. *Mesorhizobium loti* normally infects *L. japonicus* through infection threads. However, Nod factor perception mutants in an *snf1* genetic background (*nfr1-1 nfr5-2 snf1*) can be infected with or without infection threads if inoculated with wild-type or nod mutant strains, respectively (Madsen *et al.*, 2010). This can be re-created using wild-type strains. In *L. burttii* roots inoculated with *S. fredii* HH103, micro-colonies form, but infection threads abort shortly after initiation of progression. Nodules nevertheless emerge and are infected probably in the absence of epidermal infection threads. However, the absence of transcellular infection threads was not demonstrated (Acosta-Jurado *et al.*, 2016). On the other hand, *Rl* Norway, an ineffective strain, infects *L. burttii* via an infection thread-independent mechanism. Our work gives independent proof of this infection duality under natural conditions. These results provide evidence that robinioid plants have an inherent ability to support different types of infection. To our knowledge, this has not been described in other legume clades.

### Epidermal infection

Crack-entry penetration of the epidermis in natural systems is often restricted to lateral or adventitious root emergence sites (Ndoye *et al.*, 1994; Subbarao *et al.*, 1995; Bonaldi *et al.*, 2011). However, in a series of *Lotus* mutants that are impaired in epidermal infection thread formation, such as *nena-1* (Groth *et al.*, 2010), *ern1-2* (Cerri *et al.*, 2017; Kawaharada *et al.*, 2017), and *rhl1* (Karas *et al.*, 2005), nodules are infected via epidermal cracks throughout the whole root. In a similar fashion, *Rl* Norway infection sites are not located at lateral root emergence sites or between intact epidermal cells (Supplementary Fig. S1a). Very often bacteria accumulated on top of empty nodule primordia, the formation of which most probably creates natural openings on the epidermis.

*Rl* Norway induces widespread root hair deformation, but no infection threads (Fig. 2). The absence of epidermal infection threads in *Rl* Norway-inoculated roots is supported by the reduced induction of *NIN*, *ERN1*, and *EPR3* at 3 dpi (Fig. 3). Moreover, the absence of cortical infection threads correlates with the reduced induction of *SYMREM1* at 14 dpi (Fig. 3), which is essential for efficient infection thread progression (Lefebvre *et al.*, 2010; P. Liang *et al.*, 2018). Recently, SYMREM1 has been shown to mediate the formation of a specific symbiotic perception hub and regulate the stability of the NFP receptor (P. Liang *et al.*, 2018). Induction of *NIN* in *Rl* Norway-treated roots was also reduced at 7 dpi. However, at 14 wpi, it reached slightly higher levels. This correlates with the appearance of the first nodule primordia. In conclusion, the microscopy and molecular evidence support an infection thread-independent crossing of the epidermis.

### Perception at the cell entry interface

‘Peg’-like structures have been described in *Lupinus albus* (Gonzalez-Sama *et al.*, 2004), *Aeschynomene afraspera* (Bonaldi *et al.*, 2011), and *Lotus* mutants (Madsen *et al.*, 2010). They resemble enlarged and deformed infection threads that arise from intercellular bacterial accumulations. By inducing spontaneous nodulation in *L. burttii*, we could assess the role of Nod factor in their formation. Nod factor synthesis is essential for the formation of these structures, as no ‘peg’-like structure was observed upon inoculation with *Rl* NorwayΔ*nodC*. Bacteria nevertheless colonized nodule cells at a very low frequency. This remaining colonization is unlikely to be caused by residual Nod factor synthesis, as the *nodC* mutant induced no root hair deformation, a sensitive Nod factor response (Supplementary Fig. S4d). It is tempting to speculate that there is a Nod factor-independent entry mechanism, as has been previously postulated (Madsen *et al.*, 2010). However, we cannot discard the possibility that by activating symbiotic signalling through SYMRK overexpression, we bypassed Nod factor signalling. Differences in the dependency of Nod factor for the formation of ‘peg’-like structures in *L. japonicus* Gifu *nfr1-1 nfr5-2 snf1* and in *L. burttii* overexpressing SYMRK could be caused by induction of an alternative signalling pathway in the latter. However, we cannot exclude that the observed effect is due to host plant differences.

The Nod factor-dependent formation of ‘peg’-like structures supports the existence of a perception checkpoint prior to cell entry. In *Medicago truncutula*, the NFP and LYK3 receptors accumulate in a narrow zone at the border between the meristematic and the infection zones (Moling *et al.*, 2014). Down-regulation of NFP impairs release of bacteria (Moling *et al.*, 2014). Moreover, Nod factors accumulate strongly in the pre-fixation zone, specially in infection threads (Timmers *et al.*, 1998). Our results are independent support for this hypothesis.

In summary, *Rl* Norway infects *Lotus* spp. through an infection thread-independent mechanism. It penetrates nodule cells via ‘peg’-like structures, the formation of which depends on Nod factor production. This reveals that *Lotus* exhibits a dual infection pattern depending on the rhizobia that it encounters. This dual infection of *Lotus* by *M. loti* MAFF and *Rl* Norway represents an exiting opportunity to perform comparative studies of infection.

## Supplementary data

Supplementary data are available at *JXB* online.

Fig. S1. Nodule distribution on root and shoot phenotype of *Lotus burttii* upon *Rhizobium leguminosarum* Norway inoculation.

Fig. S2. Intra- and intercellular colonization of *Rhizobium leguminosarum* Norway in *Lotus burttii* root nodules.

Fig. S3. *Mesorhizobium loti* MAFF303099 and *Rhizobium leguminosarum* Norway viability in *Lotus burttii* nodules.

Fig. S4. Nod operon and phenotypes of *Lotus burttii* upon *Rhizobium leguminosarum* Norway*ΔnodC* inoculation.

Table S1. Strains and plasmids.

Table S2. PCR primer list.

Table S3. qRT–PCR primer list.

Table S4. Nod factor structures assigned from product ion mass spectra.

Supplementary Material
