# Supplementary material for: A subcompatible rhizobium strain reveals infection duality in Lotus
Source: J Exp Bot. 2019 Feb 18;70(6):1903–13. doi: 10.1093/jxb/erz057 (PMC6436148; doi:10.1093/jxb/erz057)
Supplement: Supplementary Material [file erz057_suppl_supplementary_material.pdf]

SUPPLEMENTARY DATA

Article title: A sub-compatible rhizobium strain reveals infection duality in *Lotus*.
Authors: Juan Liang, Andreas Klingl, Yen-Yu Lin, Emily Boul, Jane Thomas-Oates, and Macarena Marín.

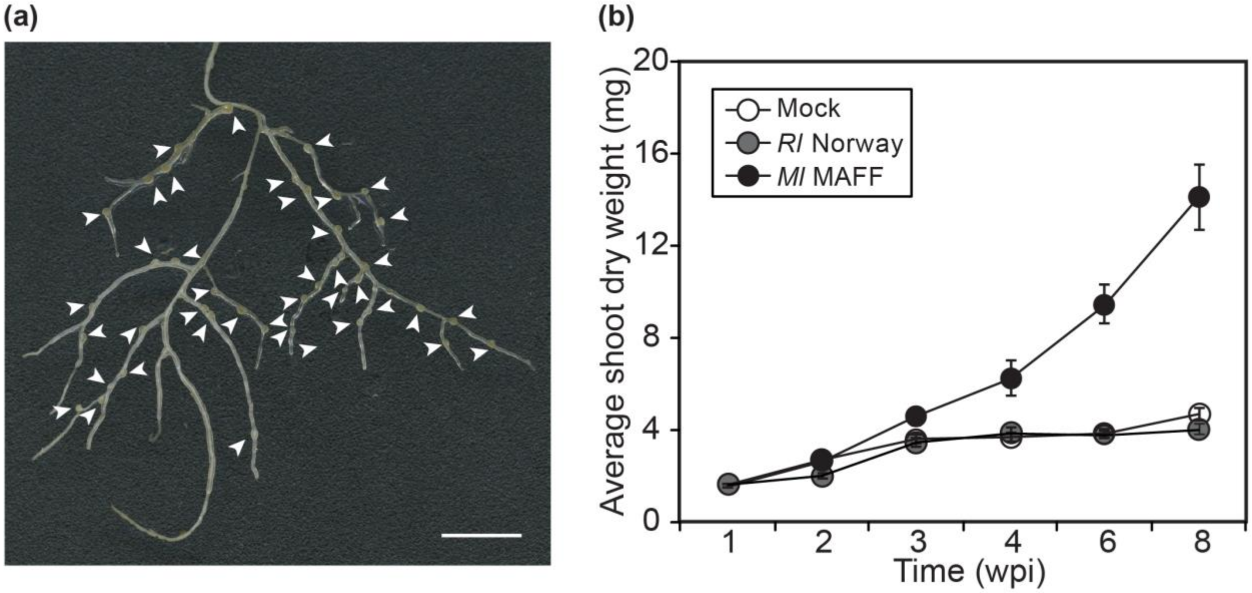

Fig. S1 Nodule distribution on root and shoot phenotype of *Lotus burttii* upon *Rhizobium leguminosarum* Norway inoculation. (a) Distribution of ineffective nodules along a representative *L. burttii* root 6 weeks after inoculation with *Rhizobium leguminosarum* Norway (*Rl* Norway). Arrowheads indicate the nodule position on the root. Bar: 1 cm. (b) Quantification of *L. burttii* average shoot dry weight upon mock treatment, and inoculation with *Rl* Norway and *Mesorhizobium loti* MAFF 303099 (*Ml* MAFF). The graph represents one of three independent experiments that were conducted with 20 plants per condition and per time point. Error bars indicate standard deviations.

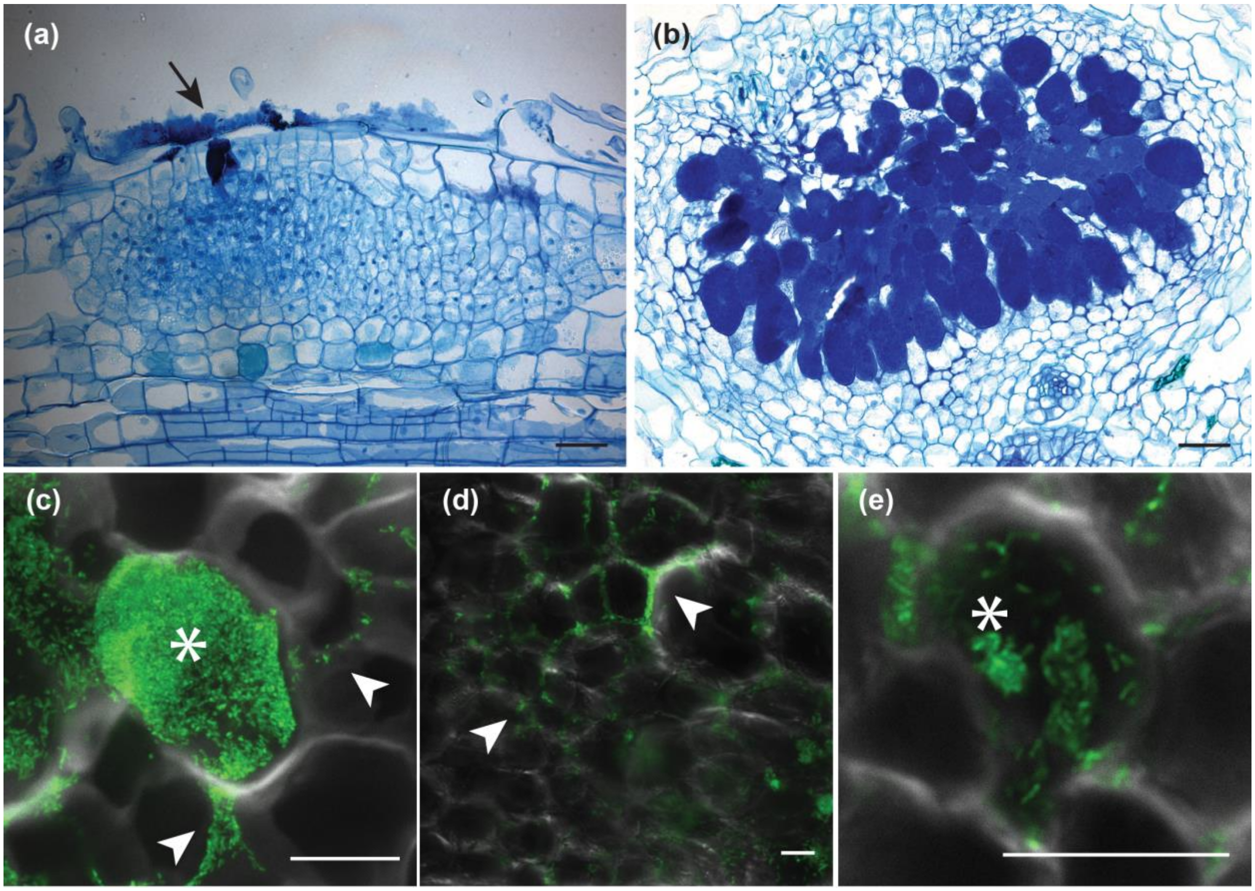

Fig. S2 Intra- and inter- cellular colonisation of *Rhizobium leguminosarum* Norway in *Lotus burttii* root nodules. Light micrographs of thin sections (5 µm) stained with toluidine blue and methylene blue show that *Rl* Norway induces the formation of infection pockets (a; arrow) and colonises in the cortex in the absence of both epidermal and trans-cellular infection threads (a, b). Enlarged cells are stained dark blue. This intense coloration is often associated with senescing cells (Van de Velde *et al.*, 2006; Regus *et al.*, 2017). Representative CLSM micrographs of nodule sections (50 µm) stained with calcofluor white (white) show that *Rl* Norway-GFP bacteria colonise nodules intra- (c, e; asterisk) and inter- cellularly (c, d; arrowheads). Abundant intercellular colonization could arise from bacteria release from plant cells (Regus *et al.*, 2017). Dense bacterial accumulations reminiscent of "pegs" often form close to the cell border (e; asterisk). Bars: (a, b) 50 µm, (c-e) 10 µm.

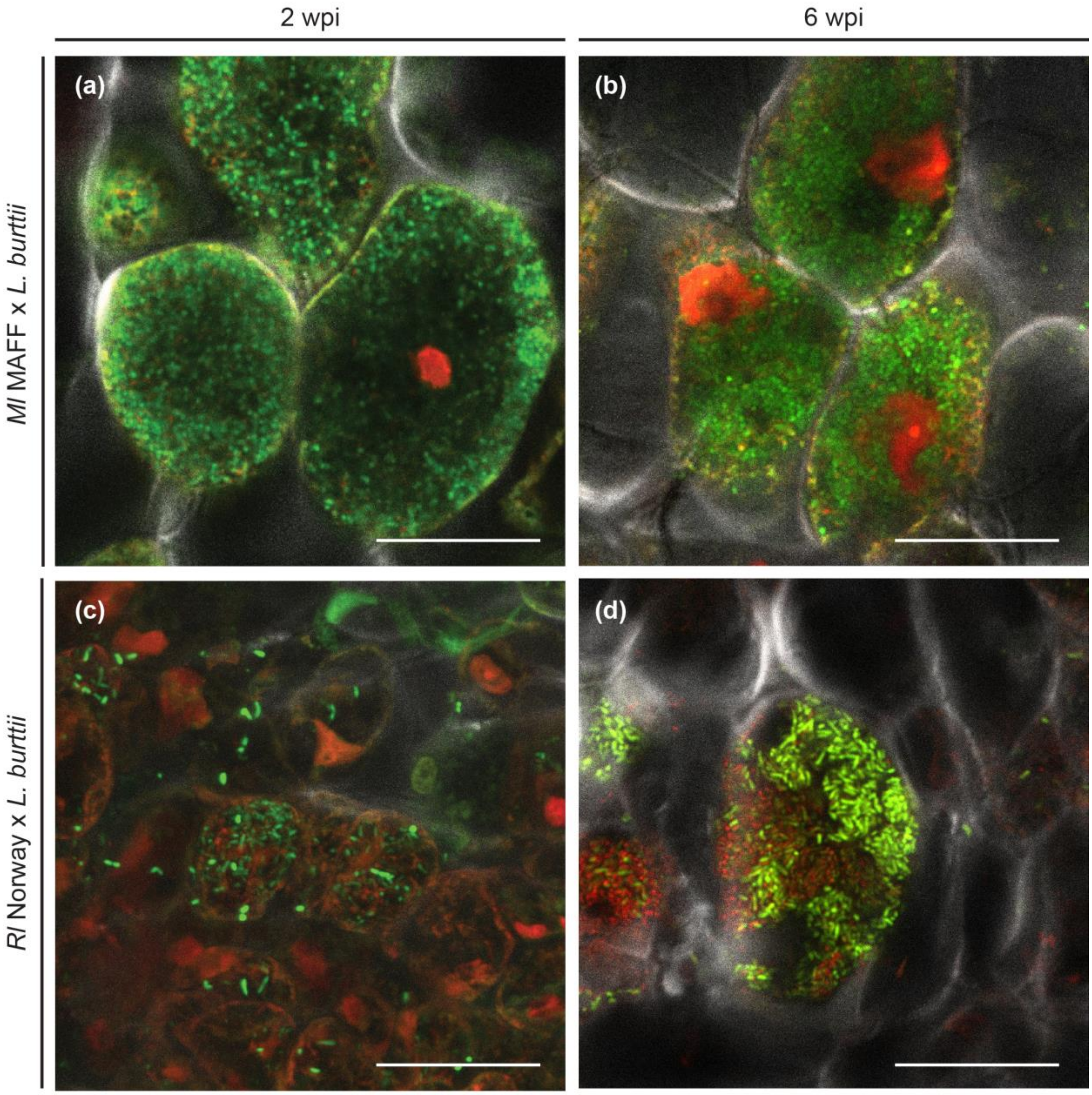

Fig. S3 *Mesorhizobium loti* MAFF303099 and *Rhizobium leguminosarum* Norway viability in *Lotus burttii* nodules. Viability was determined by live/dead staining using SYTO9 (green) and propidium iodide (red), which stain living and dead bacteria, respectively. The nodule semi-thin sections (50 µm) were counterstained with calcofluor white (white). Representative CLSM micrographs show that *Ml* MAFF bacteria are alive in nodules 2 and 4 weeks after inoculation (a, b). *Rl* Norway bacteria are alive in nodule 2 weeks post inoculation (c). However, some dead bacteria appear after 4 weeks (d). Micrographs show representative phenotypes of more than 12 nodules collected in three independent experiments. Bars: 25 µm.

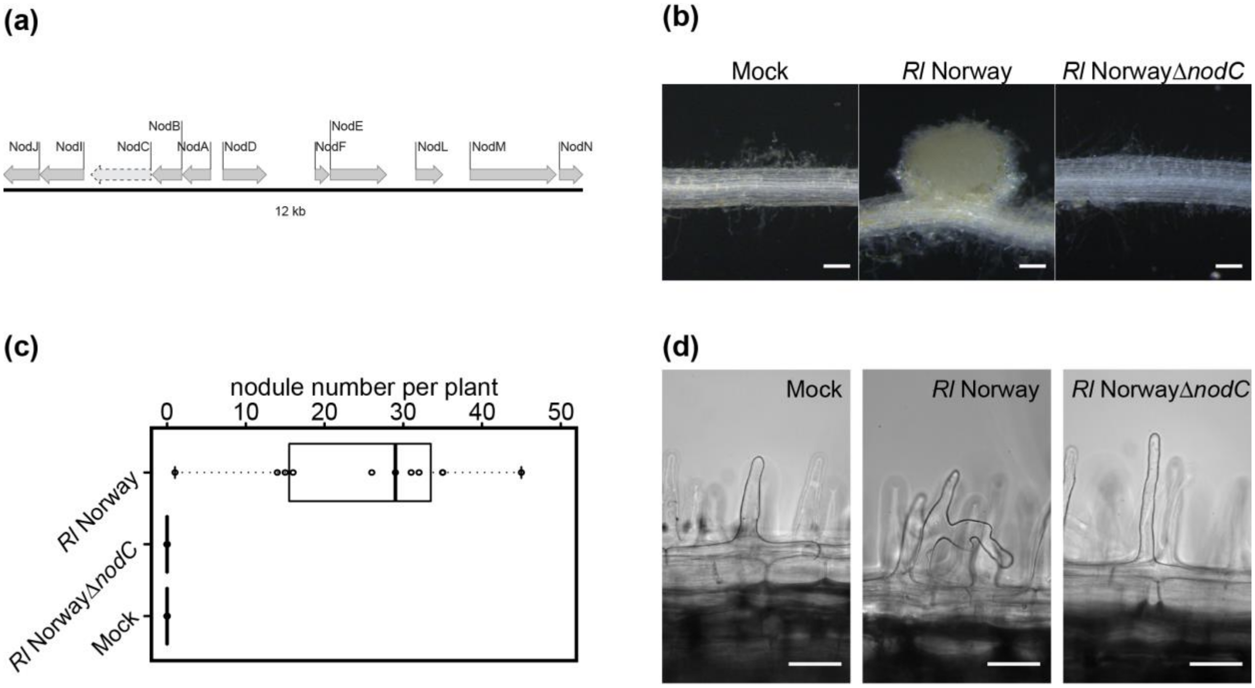

Fig. S4 Nod operon and phenotypes of *Lotus burttii* upon *Rhizobium leguminosarum* Norway*ΔnodC* inoculation. (a) The dashed box indicates the region deleted in frame by homologous recombination in the *nod* operon. Micrographs of representative root nodule phenotypes (b) and boxplot of nodule number quantification (c) 4 weeks post inoculation. Representative root hair responses to mock treatment, and inoculation with *Rl* Norway and the *Rl* NorwayΔ*nodC* mutant at 2 wpi (d). Bars: (b) 100 µm; (d) 50 µm.

Table S1 Strains and plasmids

**Strain or plasmid**	**Derivation and relevant genotype**	**Reference**
Rhizobium leguminosarum		
Norway	Wild type	(Gossmann *et al.*, 2012)
Norway GFP	*R. leguminosarum* Norway containing the pHC60 plasmid, IncP, Tc^R	(Gossmann *et al.*, 2012)
Norway Sm^R	Spontaneous Sm^R mutant of *R. leguminosarum* Norway, Sm^R	This work
NorwayΔ*nodC Ds*Red	*NodC* deletion of *R. leguminosarum* Norway containing pFAJ-*Ds*Red plasmid, Tc^R	This work
Mesorhizobium loti		
MAFF303099 *Ds*Red	MAFF3030999 strain expressing *Ds*Red, Gm^R	(Maekawa-Yoshikawa *et al.*, 2009)
MAFF303099 GFP	MAFF 303099 containing the pFAJ-GFP plasmid, Tc^R	This work
Agrobacterium rhizogenes		
AR1193	pRi1193 carrying pBR322 in the TL segment, Rf^R, Cm^R	(Stougaard *et al.*, 1987)
Escherichia coli		
TOP10	F- *mcrA* Δ(*mrr-hsd*RMS-*mcr*BC) Φ80*lac*ZΔM15 Δ *lac*X74 *rec*A1 *ara*D139 Δ(*araleu*)7697 *gal*U *gal*K *rps*L *end*A1 *nup*G, Sm^R	Invitrogen
ST18	S17 λpir Δ*hemA*, Tp^R, Sm^R	(Thoma & Schobert, 2009)

Plasmids		
pFAJ-GFP	pFAJ1708 carries the GFP encoding gene, Tc^R	(Kelly *et al.*, 2013)
pK19MOBSACB	Integration vector with the ColE1 replication origin, *mob*, *sacB*, *lacZα*, Km^R	(Schäfer *et al.*, 1994)
pK19MOBSACB- *nodC*-AB	pK19MOBSACB derivative carrying upstream 637bp and downstream 631bp flanking fragments of *nodC* (downstream of *nodJ* and *nodL*) regions, Km^R	This work
pUBi:SYMRK-mOrange	Assembled by BpiI cut ligation from: LII dy 1-2 + LII F 2-3 *pUBi:SYMRK:mOrange* + LII dy 3-4 + LII F 5-6 *p35S:GFP* + LIII β F A-B, Km^R	(Ried *et al.*, 2014)

Tp, trimethoprim; Sm, streptomycin; Tc, tetracycline; Km, kanamycin; Gm, gentamicin; Cb, carbenicillin; Rf, rifampicin.

Table S2 PCR primer list

Primers	Sequence (5’-3’)
M13_Fwd	TGTAAAACGACCCCCCAGT
M13_Rev	GGAAACAGCTATGACCAT
nodC_FrA_F	GGGAAGCTTCAGAATGAGTAGCTGCGG
nodC_FrA_R	ATGCTCTCCACCGTTTACGCATATAGTGGCGAGTGATGATCGC
nodC_FrB_F	GTAAACGGTGGAGAGCAT
nodC_FrB_R	CCCTCTAGACTAATCCATTCTGCACGCC
nodC_outer_F	TGGGTCGTTAGAAGAATTGT
nodC_outer_R	ATGTCCTCGTATTGGTAGT

Table S3 qRT-PCR primer list

Primers	Sequence (5'-3')	Reference
Rl_nifH_qF	TCCAAACTCATCCATTTCGT	This work
Rl_nifH_qR	AGTCCGGCGCATATTGGATCA	This work
IF-1_F	CGAAAACGAACACGAGATCA	(Garcia Angulo *et al.*, 2013)
IF-1_R	GTAGGGCGTCATTTCCACAA	(Garcia Angulo *et al.*, 2013)
nifH (Forward)	TCCAAGCTCATCCACTTCGTG	(Ott *et al.*, 2005)
nifH (Reverse)	AGTCCGGCGCATACTGGATTA	(Ott *et al.*, 2005)
Ml_IF_qF	GAAGTCCTCGAGTTTCCGGG	This work
Ml_IF_qR	TTGAAGCGGTAGGTGATGCG	This work
ERN1-314-Fw	TGTCTCCTTGGATTCCCCTC	(Cerri *et al.*, 2012)
ERN1-391-Rev	TTGGGGCAGGAACATCAACA	(Cerri *et al.*, 2012)
nin (Fw)	AACTCACTGGAAACAGGTGCTTTC	(Kumagai *et al.*, 2006)
nin (Rev)	CTATTGCGGAATGTATTAGCTAGA	(Kumagai *et al.*, 2006)
Ljnpl qF	CCACATTGCTGGAGGGCCTTG	(Xie *et al.*, 2012)
Ljnpl qR	GCTCACGTACCCACTGCCAC	(Xie *et al.*, 2012)
epr3 (Fw)	TGGCAGCAGTTTTGAACAAG	(Kawaharada *et al.*, 2015)
epr3 (Rev)	GTCTTCAGCGGGGTATTTGA	(Kawaharada *et al.*, 2015)
ATP (Fw)	CAATGTCGCCAAGGCCCATGGTG	(Kawaharada *et al.*, 2015)
ATP (Rev)	AACACCACTCTCGATCATTTCTCTG	(Kawaharada *et al.*, 2015)

Table S4 Nod Factor structures assigned from product ion mass spectra

Strain	Structures
R. leguminosarum Norway	IV(16:1-OH)
	IV(16:1, Ac)/(C18:2-OH)
	IV(C18:4, Ac)
	IV(C18:3, Ac)
	IV(18:1, Ac)
	IV(18:0, Ac)
	IV(C18:1-OH, Ac)
	IV(C20:4, Ac)
	IV(C20:1, Ac)
	IV(C20:4-OH, Ac)
	V(C16:1-OH)
	V(18:1)
	V(C16:1, Ac)/(C18:2-OH)
	V(C16:1-OH, Ac)/(C20:0)
	V(C18:4, Ac)
	V(18:1, Ac)/(C20:2-OH)
	V(C18:0, Ac)(C20:1-OH)
	V(C20:3,Ac)
	V(C20:1, Ac)

References

Cerri MR, Frances L, Laloum T, Auriac MC, Niebel A, Oldroyd GE, Barker DG, Fournier J, de Carvalho-Niebel F. 2012. *Medicago truncatula* ERN transcription factors: regulatory interplay with NSP1/NSP2 GRAS factors and expression dynamics throughout rhizobial infection. *Plant Physiology* **160**(4): 2155-2172.

Garcia Angulo VA, Bonomi HR, Posadas DM, Serer MI, Torres AG, Zorreguieta A, Goldbaum FA. 2013. Identification and characterization of RibN, a novel family of riboflavin transporters from *Rhizobium leguminosarum* and other proteobacteria. *Journal of Bacteriology* **195**(20): 4611-4619.

Gossmann JA, Markmann K, Brachmann A, Rose LE, Parniske M. 2012. Polymorphic infection and organogenesis patterns induced by a Rhizobium leguminosarum isolate from *Lotus* root nodules are determined by the host genotype. *New Phytologist* **196**(2): 561-573.

Kawaharada Y, Kelly S, Nielsen MW, Hjuler CT, Gysel K, Muszynski A, Carlson RW, Thygesen MB, Sandal N, Asmussen MH, et al. 2015. Receptor-mediated exopolysaccharide perception controls bacterial infection. *Nature* **523**(7560): 308-312.

Kelly SJ, Muszynski A, Kawaharada Y, Hubber AM, Sullivan JT, Sandal N, Carlson RW, Stougaard J, Ronson CW. 2013. Conditional requirement for exopolysaccharide in the *Mesorhizobium-Lotus* symbiosis. *Molecular Plant Microbe Interactions* **26**(3): 319-329.

Kumagai H, Kinoshita E, Ridge RW, Kouchi H. 2006. RNAi knock-down of *ENOD40s* leads to significant suppression of nodule formation in *Lotus japonicus*. *Plant and Cell Physiology* **47**(8).

Maekawa-Yoshikawa M, Müller J, Takeda N, Maekawa T, Sato S, Tabata S, Perry J, Wang TL, Groth M, Brachmann A, et al. 2009. The temperature-sensitive brush mutant of the legume *Lotus japonicus* reveals a link between root development and nodule infection by rhizobia. *Plant Physiology* **149**(4): 1785-1796.

Ott T, van Dongen JT, Gunther C, Krusell L, Desbrosses G, Vigeolas H, Bock V, Czechowski T, Geigenberger P, Udvardi MK. 2005. Symbiotic leghemoglobins are crucial for nitrogen fixation in legume root nodules but not for general plant growth and development. *Current Biology* **15**(6): 531-535.

Regus JU, Quides KW, O'Neill MR, Suzuki R, Savory EA, Chang JH, Sachs JL. 2017. Cell autonomous sanctions in legumes target ineffective rhizobia in nodules with mixed infections. *American Journal of Botany* **104**(9): 1-14.

Ried MK, Antolin-Llovera M, Parniske M. 2014. Spontaneous symbiotic reprogramming of plant roots triggered by receptor-like kinases. *Elife* **3**.

Schäfer A, Tauch A, Jäger W, Kalinowski J, Thierbach G, Pühler A. 1994. Small mobilizable multi-purpose cloning vectors derived from the *Escherichia coli* plasmids pK18 and pK19: selection of defined deletions in the chromosome of *Corynebacterium glutamicum*. *Gene* **145**(1): 69-73.

Stougaard J, Abildsten D, Marcker KA. 1987. The *Agrobacterium rhizogenes* pRi TL-DNA segment as a gene vector system for transformation of plants. *Molecular and General Genetics* **207**(2-3): 251.

Thoma S, Schobert M. 2009. An improved *Escherichia coli* donor strain for diparental mating. *FEMS Microbiology Letters* **294**: 127–132.

Van de Velde W, Guerra JC, De Keyser A, De Rycke R, Rombauts S, Maunoury N, Mergaert P, Kondorosi E, Holsters M, Goormachtig S. 2006. Aging in legume symbiosis. A molecular view on nodule senescence in *Medicago truncatula*. *Plant Physiology* **141**(2): 711-720.

Xie F, Murray JD, Kim J, Heckmann AB, Edwards A, Oldroyd GE, Downie JA. 2012. Legume pectate lyase required for root infection by rhizobia. *Proceedings of the National Academy of Sciences* **109**(2): 633-638.
